# Concurrent Validity of Dual-Task Walking Speed With CERAD-NP Assessment Battery in Community-Dwelling Older Adults

**DOI:** 10.1016/j.arrct.2023.100291

**Published:** 2023-08-12

**Authors:** Han suk Lee, Mansoo Ko, Hyung-Ji Kim

**Affiliations:** a Department of Physical Therapy, Eulji University, Seongnam-si, Gyeonggi-do, South Korea; b Department of Physical Therapy, University of Texas Medical Branch, Galveston, TX, USA; c Department of Neurology, Uijeongbu Eulji Medical Center, Eulji University School of Medicine, Uijeongbu, South Korea

**Keywords:** Alzheimer, CERAD-NP Assessment Battery Cognition, Dual-task, Walking

## Abstract

**Objective:**

To explore the concurrent validity of the dual-task walking speed assessments in older adults using the Consortium to Establish a Registry for Alzheimer's Disease Neuro-Psychological (CERAD-NP) Assessment Battery.

**Design:**

Cross-sectional design.

**Setting:**

Welfare care centers, Senior complex centers, and Dementia prevention care centers.

**Participants:**

A total of 163 community-dwelling older adults (N=163) were recruited using consecutive sampling. Participants were composed of 65 older adults with cognitive decline and 98 without cognitive decline.

**Interventions:**

Not applicable.

**Main Outcome Measures:**

This study assessed the concurrent validity between dual-task walking speed assessments and the Total II score of CERAD-NP using Spearman's rank order correlations. The effect of the dual-task walking speed assessments on the Total II score was further investigated through multiple linear regression analysis.

**Results:**

There was a moderate and statically significant association between the Total II score and all 8 dual-task walking speed assessments (*P*<.05). The Total II score was strongly associated with the dual tasks of walking on a straight path while counting backward and crossing over an obstacle (*r*=0.698, *r*=0.697, respectively; *P*<.05). According to multiple linear regression, only the dual task of walking while counting backward was significantly associated with the Total II score (*P*<.05).

**Conclusion:**

The dual-task walking speed assessments, which involved walking and performing a secondary task such as counting backward or crossing an obstacle on a straight path, were highly indicative of cognitive decline. The combination of results from both tasks may provide a more comprehensive evaluation of cognitive decline compared with relying solely on a single-task assessment.

Regions of the brain involved in walking overlap with areas that play a role in cognitive processes.^1^^,^^2^ The degeneration or damage caused by normal aging and certain diseases such as dementia and Parkinson's disease has a significant effect on these brain regions, leading to a decline in cognitive abilities and deviations in walking performance.^3^^,^^4^ Clinically, severe gait deviations in older adults could serve as a biobehavioral indicator of cognitive impairment. This is because gait performance includes cognitive processes such as attention, memory, perception, and decision-making, which are essential for maintaining balance, coordinating movements, and avoiding obstacles while walking.^1^^,^^2^ Previous studies indicated that concurrent performance of various tasks, such as motor (eg, carrying a glass of water) and cognitive tasks (eg, counting backward), may cause gait deviations.^5^, ^6^, ^7^, ^8^ Bishnoi and Hernandez^9^ showed that serial subtraction and verbal frequency are effective tasks when combined with a gait performance task for detecting mild cognitive impairment (MCI) related changes. Under dual-task conditions, gait velocity, stride time, and gait variability were significantly deviated compared with a single-task test in individuals with cognitive decline.^10^ Additionally, the complexity of walking on a curved path has a significant effect on stability, balance, and coordination, leading to deviations from individuals’ normal gait patterns.^11^ Furthermore, performing multiple tasks simultaneously may require elevated cortical activity and, thus, a greater cognitive effort than performing simple tasks alone.^12^ Thus, the ability to perform a secondary task while walking may serve as a potential screening tool for cognitive decline in older adults.

Montero-Odasso et al^13^ and Sobol et al^14^ have reported that cognitive function is associated with walking speed when older adults also perform a cognitive dual task, such as counting backward. Wittear et al^15^ and Román et al^12^ found that walking speed while performing a motor dual task, such as carrying a tray, walking on steps, or crossing over obstacles, was strongly associated with cognitive function. Despite these findings, the previous studies have not taken into account the type of walking path. In daily life, individuals encounter a variety of walking paths, such as navigating around a curved path, for instance, walking around the corner of a building. As a result, dual-task walking assessments should also assess walking performance in older adults while traversing a nonlinear walking path. Currently, little attention has been paid to the standard assessments of dual-task walking speed, particularly when it comes to incorporating nonlinear walking paths. Given the significant effect of cognitive function on gait performance, it is imperative to prioritize the development and validation of a standardized dual-task walking speed assessment that incorporates both linear and nonlinear walking paths. This would greatly enhance our understanding of the relation between cognitive function and walking ability in older adults and facilitate more effective evaluation and treatment of gait deviations.

The Consortium to Establish a Registry for Alzheimer's Disease NeuroPsychological (CERAD-NP) assessment battery was developed for patients with Alzheimer disease^16^ and has been widely used as a tool for dementia screening.^17^ The CERAD-NP has been used to detect cognitive decline in older adults at national dementia care centers in Korea, which manage the older population at the national level,^17^^,^^18^ but as an assessment tool, it is expensive and time-consuming. On the other hand, a walking speed test is a simple, cost-effective, professional, and independent tool appropriate for use in clinical settings. Dual-task walking speed assessments have been a feasible approach for clinicians to assess cognitive decline, but these tests have not been validated by gold-standard cognitive tests to determine the severity of cognitive impairments. This study aimed to examine the concurrent validity of dual-task walking speed assessments compared with a well-established CERAD-NP in older adults. We hypothesized that there would be a strong positive association between dual-task walking speed assessments and the Total II score of CERAD-NP. Thus, older adults with lower total II scores on the CERAD-NP would exhibit slower dual-task walking speed compared with those with higher scores on the CERAD-NP.

## Methods

### Participants

A total of 65 older adults with cognitive decline and 98 without cognitive decline were recruited from local communities. Table 1 shows the general demographic characteristics of the participants. We categorized the study participants into 2 distinct groups based on their cognitive functioning. The first group consisted of cognitively normal individuals (n=98), while the second comprised those experiencing cognitive decline (n=65).

**Table 1:** Participant characteristics (n=163)

Variables (Unit)		Without Cognitive Decline (n=98)	With Cognitive Decline (n=65)	*P*
Age (year)		76.1 (5.7)	79.6 (5.6)	<.001*
Education level (year)		8.4 (4.8)	5.5 (4.4)	<.001*
Sex Women n (%)		77/98 (78.6%)	49/65 (75.4%)	.637
BMI (kg/m^2^)		23.7 (2.6)	24.4 (3.1)	.162
MMSE (score)		25.6 (3.6)	18.1 (6.3)	<.001*
Total Ⅱ (score)		70.5 (13.8)	42.3 (16.2)	<.001*
Walking speed (m/s)		0.9 (0.3)	0.6 (0.2)	<.001*
Comorbid condition	DM n (%)	16/98 (16.3%)	19/65 (29.2%)	
	HBP n (%)	35/98 (35.7%)	30/65 (46.2%)	
	HLD n (%)	29/98 (29.6%)	22/65 (33.8%)	

NOTE. Values are mean (Standard deviation) unless otherwise indicated.

Abbreviations: BMI, body mass index; DM, diabetes mellitus; Edu, education; HBP, high blood pressure; HLD, hyperlipidemia; LBP, low back pain; MMSE, Mini-Mental State Examination; n, number; SS, spinal stenosis.

⁎ *P*<.05.

The cognitively normal participants were initially identified from a larger cohort of older adults without any prior cognitive impairment diagnoses. The selection process began with a preliminary screening using the Mini-Mental State Examination (MMSE), with a focus on individuals who scored 24 or above. To ensure the accuracy of their cognitive status, we further used the CERAD-Assessment Packet. This ensured the participants met the required criteria for being considered cognitively normal. The process was made even more robust by a secondary verification by a clinical psychologist and a psychiatrist, reinforcing the validity of our classification.

In the case of participants with cognitive decline, the Mini-Mental State Examination (MMSE) was similarly employed as the initial screening tool. Here, our focus was on individuals who scored 24 or below. We also used the CERAD-Assessment Packet to provide an additional layer of verification for their cognitive status, ensuring they met the criteria for the cognitive impairment category. As with the cognitively normal participants, the accuracy of this categorization was further enhanced by a subsequent evaluation conducted by both a clinical psychologist and a psychiatrist. This rigorous dual verification process aimed to ensure the credibility of our classifications.

A cross-sectional study with consecutive sampling was used to recruit older adults from the following facilities in Gyeonggi-do, Korea, from October 2018 to August 2021: 2 older adults welfare care centers, 1 senior complex center, and 2 dementia prevention care centers. Of the 195 participants initially recruited, 32 dropped out for the following reasons: stroke (13), Parkinson's disease (5), a simple change in heart rhythm during the test (3), missing data on the cognitive test (3), a recent history of falls (1), dizziness (1), artificial joint surgery (1), walking instability for an unknown reason (1), epilepsy (1), and actual age <65 years (1). The inclusion criteria were (a) ability to walk independently, (b) ability to understand the instructions of the tester, and (c) age >65 years. The exclusion criteria were (a) use of a wheelchair for locomotion, (b) those who had acute injuries (eg, bone fractures), (c) those who were unable to comprehend the study consent, instructions, and follow directions of study team, and (d) legal blindness.

The participants of this study were thoroughly informed about the protocol and their written consent was obtained prior to their participation, ensuring that they fully understood the procedures involved in the study. The Institutional Review Board of Eulji University approved the study (study approval number: EUIRB2020-007).

### Measures

The Korean version of the CERAD-NP was used by a trained evaluator to measure cognitive function. The Total II score, which serves as an indicator of cognitive function, was calculated by aggregating the scores obtained from the 7 components of the CERAD-NP: verbal fluency test (maximum score 24), modified Boston naming test (maximum score 15), word list memory test (WLMT, maximum score 30), constructional praxis test (CPT, maximum score 11), word list recall test (WLRT, maximum score 10), word list recognition test (word list recognition test, maximum score 10), and constructional praxis recalls test (maximum score 11). The maximum Total II score is 111 points.^18^^,^^19^ Prior to initiating the tasks, the examiner provided a thorough explanation of the tasks, both verbally and through demonstrations, to ensure clear understanding. After ensuring that the participants fully understood the tasks, the experimental test was then conducted. The sequence of the walking tasks was predetermined, starting with the less complex straight-line walking task followed by the more demanding curved-path walking. This order was intentionally selected to minimize any learning effects that could influence the outcomes of tasks. Additionally, we ensured there were sufficient rest periods between tasks to minimize the effect of fatigue in older adults. Given these measures, we believe our study design has effectively addressed potential issues related to fatigue and learning effects. Moreover, as our research follows a cross-sectional study design rather than an intervention study, the likelihood of learning effects having a significant influence is even lower.

The trained evaluator employed a stopwatch to obtain the amount of time in which each participant took to traverse a distance at a comfortable pace. Participants performed the 8 walking tasks: walking on a straight path (baseline test) (Task 1), walking on a straight path while counting backward from 20 (Task 2), walking on a straight path while holding a cup of water (Task 3), walking on a straight path while crossing over obstacles (Task 4), walking on a curved path (Task 5), walking on a curved path while counting backward from 20 (Task 6), walking on a curved path while holding a cup of water (Task 7), and walking on a curved path while crossing over obstacles (Task 8). Participants performed the walks while looking at a visible target circle on the wall. The distance covered was marked on the walkway, and the curved path was printed on the walkway.

**Fig 1:**
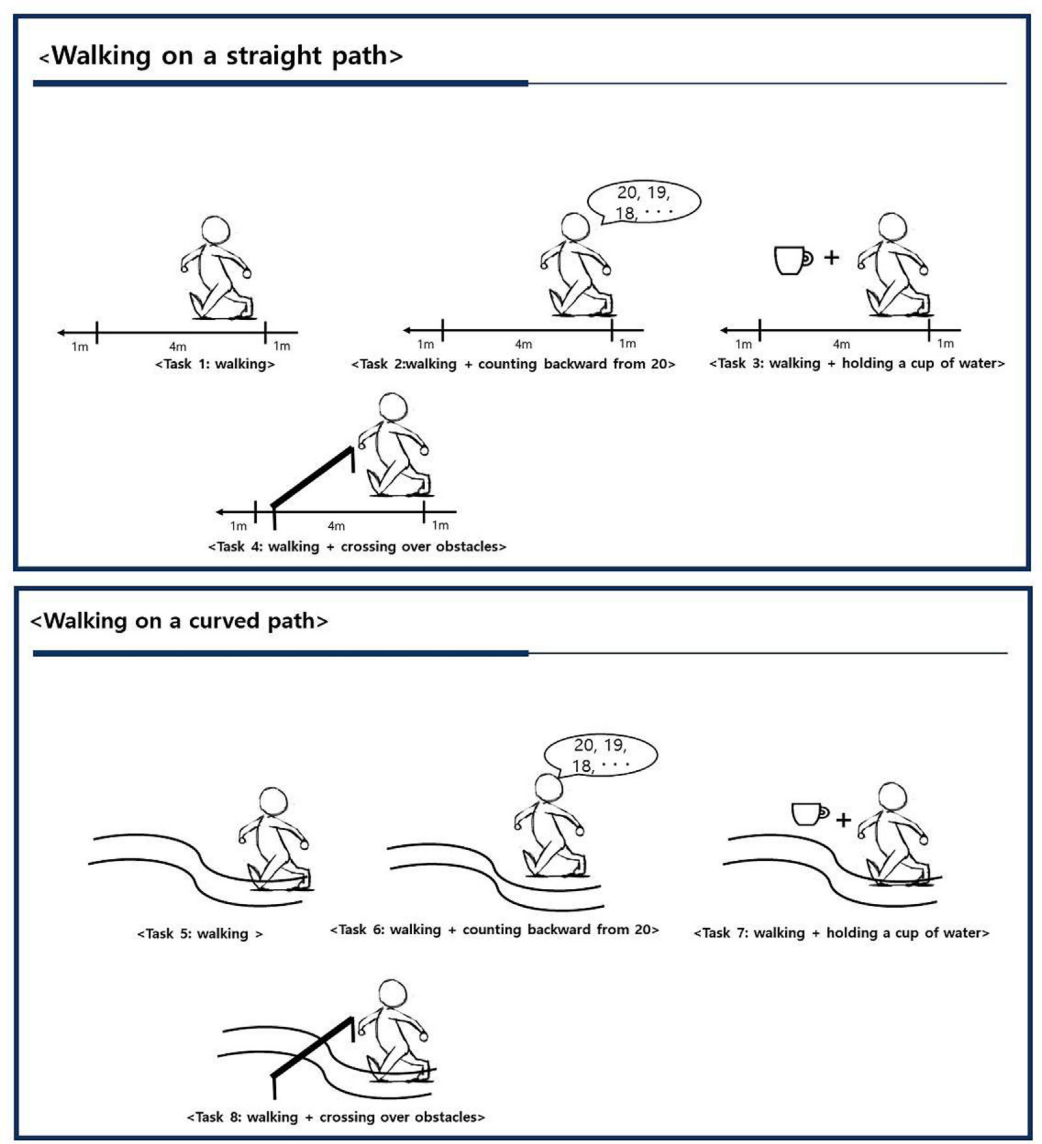
Walking with 8 different tasks.

**Fig 2:**
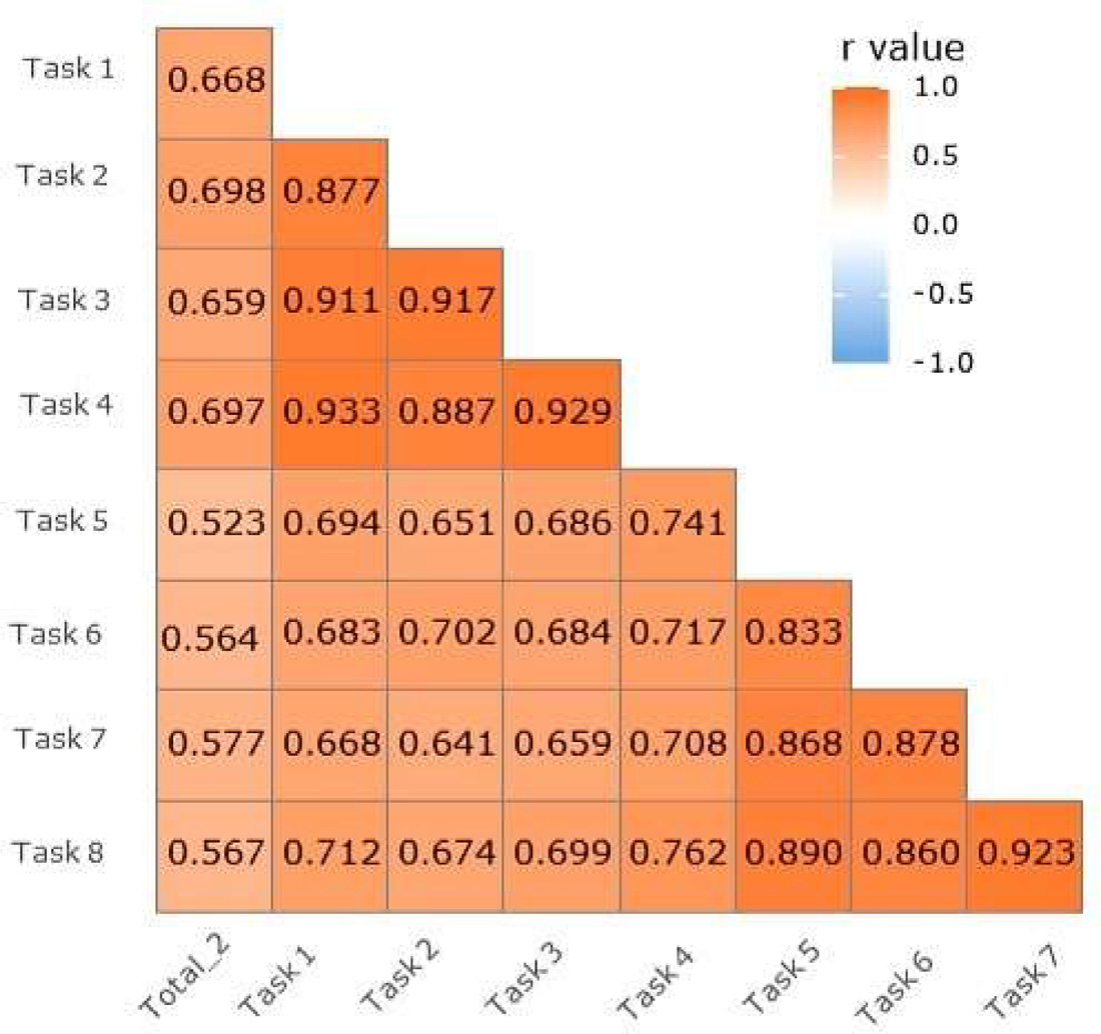
Correlation coefficients between the Total Ⅱ score and the walking speed for each dual-task walking speed assessments.

The dual-task test involving walking on a straight path (ie, Task 1 to Task 4, fig 1) used a 4-meter walking test (intraclass correlation coefficient=0.82).^20^ Both the 4-meter and the 10-meter walk test were shown as valid measures for assessing walking speed in healthy older individuals.^21^ However, for older adults experiencing cognitive deficits, the 4-meter walk test is often more feasible and practical. The 4-meter walk test is especially beneficial in clinical environments that may have space constraints, such as smaller outpatient clinics, rehabilitation centers, or nursing homes, where larger distances for walking tests may not be readily available. This allows for effective evaluation of gait performance even within these limited spaces. The dual-task test involving walking on a curved path (ie, Task 5 to Task 8, fig 2) was assessed with a Groningen Meander walking test (intraclass correlation coefficient=0.99).^20^

The walking performance speed test was the basis of the dual-task test because walking is the most common task of daily living. The dual tasks in this study consisted of 3 types of tasks: motor-motor dual tasks, motor-cognitive tasks, and complex tasks. The motor-motor dual task involved walking and simultaneously performing another motor task (ie, Task 3, Task 4, Task 5). The motor-cognitive dual task involved walking and a simultaneous cognitive task, such as counting backward (Task 2). The complex dual-task involved walking on a curved path and simultaneously performing another type of task (ie, Task 6, Task 7, Task 8, fig 1).

### Statistical analysis

Descriptive statistical tests were used to analyze the general characteristics of the participants. The Kolmogorov–Smirnov test was used to verify the normality of the distribution of continuous variables. The distributions of some variables showed normal distribution, but others did not. To evaluate the concurrent validity, Spearman's rank order correlation test was used to determine the relation between the cognitive function test (Total Ⅱ score) and the dual-task walking test (walking speed). Additionally, multiple linear regression analysis was conducted with an enter method to identify the most influential variables. The independent variables were walking speed while performing the 8 walking tasks, and the dependent variables were the TotalⅡscores. Age and education level were used as covariates in the analysis. The data were analyzed using SPSS version 21.0.^a^ All statistics were 2-tailed, and *P* values <.05 were considered significant.

## Results

The results of the study showed that there was a significant positive correlation between the Total II score and the dual-task walking speed for all 8 tasks (*P*<.05). This demonstrates the good concurrent validity of the dual-task walking speed assessments. In particular, the Total II score was strongly correlated with 2 tasks performed on a straight path, such as walking while counting backward and walking while crossing over an obstacle (*r*=0.698, *r*=0.697; *P*<.05) as shown in table 2.

**Table 2:** Correlation coefficients between the Total Ⅱ score and the walking speed for each dual-task walking test (n=163)

	Task 1	Task 2	Task 3	Task 4	Task 5	Task 6	Task 7	Task 8
Total Ⅱ	.668*	.698*	.659*	.697*	.523*	.564*	.577*	.567*

NOTE. Values are correlation coefficients.

Tasks were as follows: Task 1: walking on a straight path (baseline), Task 2: walking on a straight path while counting backward from 20, Task 3: Walking on a straight path while holding a cup of water, Task 4: Walking on a straight path while crossing over obstacles, Task 5: Walking on a curved path, Task 6: Walking on a curved path while counting backward, Task 7: Walking on a curved path while holding a cup of water, Task 8: Walking on a curved path while crossing over an obstacle.

⁎ *P*<.05.

Further analysis using multiple linear regression revealed that after controlling for age and education level, the Total II score had a significant relation with the walking test while counting backward on a straight path (β=0.350, *P*<.05) as shown in table 3. Additionally, the Spearman rank order correlation was computed to assess the relation between the dual-task walking speed of each task and the scores of the Total II subscale tests. The results showed a significant positive correlation between the Total II subscale test scores and the dual-task walking speed for all tasks (*P*<.05). Of particular interest, the WLMT showed a strong association with all dual-task walking speed results (*P*<.05), as shown in table 4.

**Table 3:** Multiple linear regression analysis of Total Ⅱ score and walking speed according to different tasks (n=163)

Predictable Variables	B	SE	β	*t*	*P*	VIF
Task 1	−2.217	12.794	−.030	−.173	.863	10.273
Task 2	24.436	10.526	.350	2.321	.022*	7.631
Task 3	−7.510	13.747	−.101	−.546	.586	11.389
Task 4	16.761	15.305	.229	1.095	.275	14.746
Task 5	−3.447	13.020	−.035	−.265	.792	5.939
Task 6	−4.414	13.913	−.046	−.317	.751	7.173
Task 7	29.449	18.300	.283	1.609	.110	10.375
Task 8	−12.409	19.947	−.118	−.622	.535	11.998
F=18.390, *P*<.05, *R*^2^=0.740, adj *R*^2^=0.518, D-W=1.391						

NOTE. Values are regression coefficients. B: Unstandardized Regression Coefficient, β: standardized Regression Coefficient.

Tasks are as follows: Task 1: walking on a straight path (baseline), Task 2: walking on a straight path while counting backward from 20, Task 3: Walking on a straight path while holding a cup of water, Task 4: Walking on a straight path while crossing over obstacles, Task 5: Walking on a curved path, Task 6: Walking on a curved path while counting backward, Task 7: Walking on a curved path while holding a cup of water, Task 8: Walking on a curved path while crossing over an obstacle.

The model was calculated using the independent variable (Walking velocity, age, education level) and dependent variable (**Total** Ⅱ **score**). Regression coefficients for age and education level are not displayed.

Abbreviations: D-W, Durbin-Watson; SE, standard error; VIF, Variance Inflation Factor.

⁎ *P*<.05.

**Table 4:** Correlation coefficients between the subscale scores of the Total Ⅱ score and walking velocity for each dual-task walking test (n=163)

Variables	VFT	MBNT	WLMT	CPT	WLRT	WLRcT	CRT
Task 1	.476*	.591*	.629*	.429*	.574*	.395*	.526*
Task 2	.478*	.626*	.646*	.496*	.615*	.381*	.627*
Task 3	.454*	.587*	.608*	.445*	.574*	.351*	.581*
Task 4	.499*	.612*	.667*	.472*	.620*	.401*	.553*
Task 5	.424*	.410*	.489*	.315*	.496*	.335*	.376*
Task 6	.470*	.505*	.493*	.340*	.504*	.335*	.448*
Task 7	.452*	.491*	.525*	.402*	.542*	.359*	.420*
Task 8	.441*	.496*	.502*	.354*	.523*	.322*	.440*

NOTE. Values are correlation coefficients.

Tasks are as follows: Task 1: walking on a straight path (baseline), Task 2: walking on a straight path while counting backward from 20, Task 3: Walking on a straight path while holding a cup of water, Task 4: Walking on a straight path while crossing over obstacles, Task 5: Walking on a curved path, Task 6: Walking on a curved path while counting backward, Task 7: Walking on a curved path while holding a cup of water, Task 8: Walking on a curved path while crossing over an obstacle.

Abbreviations: CPT, constructional praxis test; CRT, constructional praxis recall test; MBNT, modified Boston naming test; WLRcT, word list recognition test; WLRT, word list recall test; VFT, verbal fluency test.

⁎ *P*<.05.

## Discussion

This study was conducted to identify the validity of a dual-task walking test in older adults with or without cognitive decline. The main findings were that the dual-task walking speed test has good concurrent validity and the dual-task walking speed assessment with counting backward on a straight walking path is the most strong and significant assessment to determine the degree of cognitive function in older adults.

According to a scoping review by Kikkert et al,^4^ slow walking precedes cognitive decline; therefore, the degree of walking speed can be used as a meaningful marker to predict cognitive decline. In this review, all studies but 1 analyzed only a single task, leading the authors to recommend the development of a more comprehensive measure of walking analysis because a test of walking speed alone might be too simplistic. In a later meta-analysis, Yang et al reported that walking slowed while performing a dual task compared with walking with no other tasks, which suggests the dual-task walking test is a more sensitive way to screen for mild cognitive impairment.^22^ Based on these findings, we assumed that walking speed might serve a useful tool for screening for cognitive decline in older adults. Thus, the Total II score of CERAD test was selected to validate the dual-task walking speed as a feasible measure of cognitive function in older adults.

As the aging process takes place, the brain with cognitive disorders frequently experiences degeneration, characterized by white matter lesions in the prefrontal cortex. The prefrontal cortex is a critical region of the brain responsible for functional movement, including regulating movement during walking. Thus, the brain degeneration in this region may result in a slower walking speed in older adults.^3^ Thus, older adults with a cognitive disorder will also exhibit slowed walking.^2^

The simultaneous performance of walking and a secondary task among older adults may lead to a reduction in walking speed for maintaining stability and safety while walking. For example, an older adult might stop walk while also carrying a tray to maintain balance.^23^ Similar to previous studies, we found that the dual-task walking speed assessments had a moderate correlation with Total Ⅱ scores. This result suggests that dual-task walking speed might be a meaningful marker to predict cognitive decline. In a meta-analysis, Yang et al^22^ claimed that gait speed was affected by the complexity of cognitive load. They also reported that the cognitive-motor interference (CMI) of gait speed in a dual-task walking test was correlated with the scores on the Montreal Cognitive Assessment (MoCA). These authors proposed that the CMI of gait speed could be a useful screening tool for mild cognitive impairment in older adults. When a cognitive task and a motor task are performed simultaneously, the motor task performance is decreased due to the CMI, which refers to the deterioration of performance in each task.^8^ Our findings also showed a correlation between walking speed in a dual-task walking assessments and scores on the CERAD-NP Total II. MoCA and CERAD-NP are similarly used to assess the level of cognitive impairment or dementia. Accordingly, we propose that dual-task walking speed assessments, which requires a significant cognitive demand, is a valid test for detecting cognitive decline in older adults. Total Ⅱ consists of 3 parts: memory, verbal, and constructional praxis. Seven of the 8 tests in Total Ⅱ are related to memory function. Previous study indicated that the ability to walk in older adults more influenced by their executive function (such as decision making, attention control, and problem solving) rather than their physical abilities and muscle memory.^24^ Thus, emotional control, inhibition, working memory, initiation, planning, prioritization, shift, organization, and self-monitoring can play a more significant role than physical abilities to determine walking ability in older adults.^25^ The ability to manipulate information while it is being processed is known as working memory. It is composed of 4 components: a modality-free central executive function, a phonological loop, a visuo-spatial sketch pad, and an episodic buffer.^25^, ^26^, ^27^, ^28^

Counting backward, for example, requires the use of the phonological loop, which deals with sounds and language.^29^ When older adults walk and count backward at the same time, they may need to use their phonological loop of working memory. Older adults with cognitive disorders such as mild cognitive impairment, frontal lobe dementia, and Alzheimer disease were found to have a slower walking speed while counting backward, which was linked to poor working memory in their executive function. This demonstrates the importance of executive function, including working memory, in affecting walking speed in older adults with cognitive disorders.^13^^,^^14^ When considering walking performance, a more pronounced reduction in walking speed under dual-task conditions, compared with a single task, could indicate that individuals with cognitive decline might prioritize cognitive tasks over motor tasks, such as walking.^10^ This could result in a slower walking speed when dual-tasking. However, it's crucial to acknowledge that various cognitive domains can affect walking differently.^30^ For example, in mild cognitive impairment, depending on whether the subtype is amnestic or non-amnestic, different cognitive functions are primarily affected, which could influence walking patterns.^31^ Therefore, a comprehensive understanding of the specific type of cognitive decline could offer valuable insights into its potential effects on walking performance and overall mobility. In this study, it was proposed that the dual task of walking and counting backward could be a useful tool to assess cognitive decline. The results showed a moderate correlation between the Total II score (*r*=0.698), which measures cognitive function, and the walking test while counting backward on a straight path. Even after adjusting for age and education level, the correlation remained unchanged. The study supports the hypothesis that walking while counting backward is a reliable indicator of cognitive decline, regardless of age and education level. The Total II score, which measure cognitive function, was found to have a moderate correlation with the walking assessment while crossing over obstacle on both a straight path and a curved path regardless of age and educational level, but it was not found to be statically significant. This could be due to the limited sample size in this study. Sakurai et al^32^ reported that scores on both the MoCA and the Wechsler Memory Scale-Revised logical memory test were linked to a person's ability to clear an obstacle while an older adult tripped over the obstacle. The working memory processes visual input to update the body's orientation and helps to redirect the foot position to avoid the obstacle. The visual memory function plays a role in the control mechanism by providing updated information about body orientation, encoding the spatial information of objects or obstacle in the environment, and suing this information to direct limb movement. In this manner, the visual memory function contributes to the feedforward control of the limb movement, complementing the peripheral sensory inputs.^33^, ^34^, ^35^ On the other hand, episodic memory, which is a component of long-term memory, is related to the gait stride time.^36^ This study identified that the scores of the dual-task walking speed assessments that involved walking while crossing over an obstacle and counting backward on a straight path showed a correlation with the WLMT score, which evaluates episodic memory, under the Total II sub-scale. As a result, this study showed that these 2 walking speed assessments (Task 2 and Task 4) could serve as a useful measurement for evaluating declines in episodic memory.

## Study limitations

This study showed that dual-task walking speed tests performed on a straight path had a stronger association with Total II scores than the assessments performed on a curved path. Walking on a curved path requires more attention than walking on a straight path, because the change of direction requires another central-mediated command from the basal ganglia loop and control of the visuospatial field. Accordingly, the delayed perception in older adults with cognitive impairment may contribute more to the slower speed observed when walking on a curved path than on a straight path.^11^^,^^37^ This finding may suggest that total II scores may have the advantage of detecting memory deficits rather than attention/perception problems.

This study has several limitations. Firstly, the small sample size reduces the generalizability of the findings. Secondly, the study did not consider the etiology of cognitive decline among the participants, and the participants were not sub-grouped based on specific cognitive conditions such as dementia or Alzheimer disease. Additionally, the study did not account for potential deconditioning factors that may affect walking speed assessments and relied solely on walking speed as a measure of the relation between walking performance and cognitive decline.

To address these limitations, future studies should consider using larger sample sizes and incorporating an analysis of the underlying causes of cognitive decline. Furthermore, it would be beneficial to consider other clinical gait parameters such as stride time, double support time, step width, and cadence in order to gain a more comprehensive understanding of the relation between walking performance and cognitive decline.

## Conclusions

This study found that dual-task walking speed assessments have a high level of concurrent validity, particularly for assessments that involve walking on a straight path while crossing an obstacle or counting backward. This study suggests that dual-task walking speed assessment can be a cost-effective approach of evaluating cognitive impairment in older adults. Accordingly, incorporating a dual-task walking speed assessment on a straight path while crossing over an obstacle with another cognitive task, such as counting backward, can provide a more comprehensive evaluation of cognitive function. The outcome from both tasks can be combined to provide a more valid assessment of cognitive decline, as opposed to relying on a single-task assessment.

## Suppliers

a. SPSS version 21.0; IBM.
